# Spatial transcriptomic interrogation of the murine bone marrow signaling landscape

**DOI:** 10.1038/s41413-023-00298-1

**Published:** 2023-11-06

**Authors:** Xue Xiao, Conan Juan, Tingsheng Drennon, Cedric R. Uytingco, Neda Vishlaghi, Dimitri Sokolowskei, Lin Xu, Benjamin Levi, Mimi C. Sammarco, Robert J. Tower

**Affiliations:** 1 Quantitative Biomedical Research Center, Peter O’Donnell Jr. School of Public Health, University of Texas Southwestern Medical Center, Dallas, TX USA; 2 Department of Surgery, University of Texas Southwestern Medical Center, Dallas, TX USA; 3 Department of Cell Biology & Applications, 10x Genomics, Pleasanton, CA USA; 4 Department of Surgery, Tulane School of Medicine, New Orleans, LA USA

**Keywords:** Bone, Physiology

## Abstract

Self-renewal and differentiation of skeletal stem and progenitor cells (SSPCs) are tightly regulated processes, with SSPC dysregulation leading to progressive bone disease. While the application of single-cell RNA sequencing (scRNAseq) to the bone field has led to major advancements in our understanding of SSPC heterogeneity, stem cells are tightly regulated by their neighboring cells which comprise the bone marrow niche. However, unbiased interrogation of these cells at the transcriptional level within their native niche environment has been challenging. Here, we combined spatial transcriptomics and scRNAseq using a predictive modeling pipeline derived from multiple deconvolution packages in adult mouse femurs to provide an endogenous, in vivo context of SSPCs within the niche. This combined approach localized SSPC subtypes to specific regions of the bone and identified cellular components and signaling networks utilized within the niche. Furthermore, the use of spatial transcriptomics allowed us to identify spatially restricted activation of metabolic and major morphogenetic signaling gradients derived from the vasculature and bone surfaces that establish microdomains within the marrow cavity. Overall, we demonstrate, for the first time, the feasibility of applying spatial transcriptomics to fully mineralized tissue and present a combined spatial and single-cell transcriptomic approach to define the cellular components of the stem cell niche, identify cell‒cell communication, and ultimately gain a comprehensive understanding of local and global SSPC regulatory networks within calcified tissue.

## Introduction

In healthy bone, skeletal stem and progenitor cells (SSPCs) are tightly regulated to self-renew, to maintain the stem cell pool, and to differentiate to replenish the population of bone-forming osteoblasts.^1,2^ Disruption of this tight SSPC regulation is associated with the onset of metabolic bone disorders leading to progressive bone loss, such as osteoporosis. Disruption of SSPC regulation is typically caused by localized changes within the bone marrow niche.^3^ Thus, identifying and characterizing the SSPCs within their local regulatory environment is an area of intense research.

While single-cell RNA sequencing (scRNAseq) has become widely used and can interrogate each individual cell within a tissue, the requirement for dissociation, often through enzymatic digestion, not only alters the transcriptional profile of each cell but also removes the ability to place these cells within their native environment. The ability to interrogate the spatial profile of these cells relative to each other in tissue only recently came about with the development of the spatial transcriptomics platform. While this technique is easily applied to soft tissue, the need to decalcify bone, a process that typically destroys mRNA, has remained an obstacle for long bone samples. As a result, scRNAseq has been the primary approach to dissect SSPC populations in long bone.^4–7^ A variety of markers have been proposed using transgenic mice and immunohistochemistry to label SSPC populations, including platelet-derived growth factor receptor alpha (PDGFRα) and SCA1 (termed PαS cells),^8^ leptin receptor (LEPR), and CXCL12 (termed CXCL12-abundant reticular cells, CAR cells).^9,10^ The application of scRNAseq has significantly advanced our understanding of the intrinsic signaling mechanisms in these SSPC populations.^5,11,12^ However, single-cell studies have revealed that previously proposed markers label discrete stromal cell subtypes, supporting the concept of heterogeneity within the proposed SSPC populations. These studies have also shown that previously identified stromal cells have expression profiles that overlap with markers for committed adipocyte lineage cells, such as *Adipoq*, and that these SSPC markers may also label progenitor cells already primed to the osteogenic and adipogenic lineages.^4,7^ This lack of consensus highlights the complexity of stem cell biology within the bone and underscores the need to better understand the mechanisms driving this diversity.

The finding that SSPC populations have multiple transcriptional profiles may be due to various factors. One possibility is that their transcriptional profile can be dictated by their localization within the marrow, a spatial interrogation difficult to assess by scRNAseq. Stem cells are regulated by the secretion of soluble factors by neighboring cells that comprise the stem cell niche. Subtle shifts in the spatial location or changes in the cell‒cell interactions of SSPCs and niche-forming marrow resident cells can have a profound impact on SSPC fate and function.^13,14^ However, understanding these spatial relationships in vivo in an unbiased manner has proven challenging. Researchers have applied large-scale scRNAseq to multiple cell types in an attempt to computationally reconstruct the stem cell niche and assign primary regulatory factors to their key cell types of origin.^12^ However, in addition to lacking any spatial information, this technique is limited in disentangling cell‒cell communication due to numerous secreted factors that are derived from the diverse cell types present within the bone marrow environment. As such, a more comprehensive understanding of the in situ SSPC niche microenvironment that places these cells in the spatial context of major niche modulators, such as the bone surface or blood vessels, is needed.^4^

Spatial transcriptomics bypasses the need to generate single-cell suspensions, preventing the introduction of potential artifacts by enzymatic digestion and providing unbiased transcriptional profiling in vivo. While widely used on soft tissues such as the brain^15,16^ or to unravel heterogeneity and spatial organization in tumors,^17,18^ this technique has only recently begun to be utilized in the context of musculoskeletal tissue. Early studies used spatial transcriptomics in soft tissue such as tendon^19,20^ or muscle^21,22^ and, most recently, we utilized this technique for mineralized bone tissue such as the developing calvarial suture^23^ or regenerating digit tip.^24^ Barring the need for tissue decalcification while still preserving mRNA in long bones, limitations in spatial resolution remain a major drawback given that each spatial spot within the Visium spatial gene expression system from 10 × Genomics represents 55 µm. Thus, a single spatial spot can encompass numerous diverse cell types in tissues with high degrees of cellularity and heterogeneity, making it challenging to determine whether changes in gene expression are the result of changes in cell composition within each spot, changes in gene expression within the cells comprising each spatial spot, or a combination of the two. In the context of intact musculoskeletal tissue, this limitation can be resolved (1) if the tissue area represents relatively low cellularity in which each spatial spot would correspond to only a few cells or (2) if cells of similar origin and transcriptome are present within each spot. However, with more heterogeneous tissue, in which cell types of very different lineages coexist within a tightly confined space, these spots can encompass tens of cells with highly unique transcriptional profiles, making it difficult to ascertain the cause of observed transcriptional changes. The bone marrow stem cell niche is a prime example of this type of composition, where cells derived from vascular, hematopoietic, and mesenchymal lineages exist within tight spatial confines, carefully regulating each other through direct and indirect signaling mechanisms.^13,14^ Taken together, the technical difficulties and tissue composition of the bone marrow niche have made it a difficult target for spatial transcriptomics.

To overcome the spatial limitations outlined above, we combined spatial transcriptomic data with bone marrow scRNAseq and several different predictive modeling packages to deconvolve the larger spatial spots into their cellular constituents. Using this approach, we first spatially localized SSPC subtype populations previously identified using scRNAseq.^5,7^ Using correlative analyses, we then mapped out cellular subtypes enriched within these SSPC-containing spatial spots, with *Pdgfra*^+^*Sca1*^+^ SSPCs preferentially localizing to the periosteal surface and *Cxcl12*^+^*Lepr*^+^ SSPCs enriching within the marrow. Finally, we used cell‒cell interaction analyses, spatial gene expression, and spatial-time analyses^23,24^ to reveal signaling networks utilized within the niche and across microdomains within the bone. This study overcomes technical and analytical obstacles for spatial transcriptomics in long bone to assess, for the first time, changes in SSPC regulation within its in vivo context.

## Results

### Analysis of adult mineralized bone by spatial transcriptomics

**Fig. 1:**
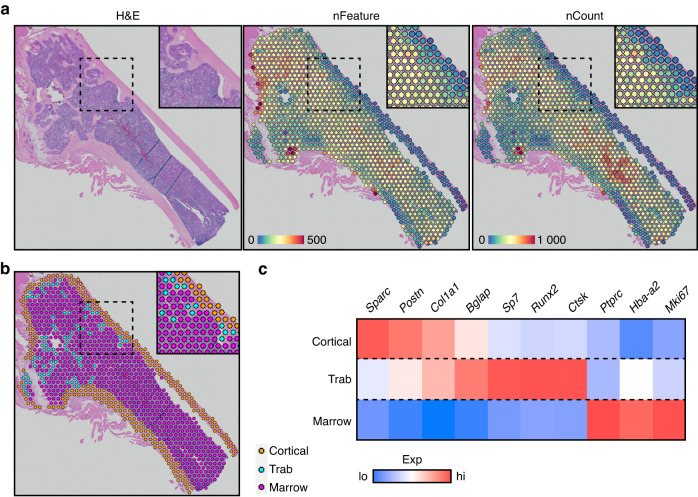
Spatial transcriptomic analyses of adult femurs. **a** Spatial feature plots of H&E-stained sections overlaid with the number of unique genes (nFeature) or unique transcripts (nCount) per spatial spot. **b** Manual segmentation of cortical bone, trabecular bone, and bone marrow spatial spots by histological morphology. **c** Marker genes showing enriched expression within each segmented compartment

Although recent advances in scRNAseq have allowed transcriptional interrogation of cells within the bone marrow, technical and analytical approaches have a limited ability to place these cellular changes within the context of their native bone environment. To overcome this limitation, we subjected adult mouse femurs to spatial transcriptomic analyses (Fig. 1). Femurs were bisected and fixed in buffered formalin overnight at 4 °C. The samples were then decalcified in EDTA for 2 weeks with fresh decal added every 2 days. The decalcified femurs were then processed for standard paraffin embedding. Histological sections were placed on the Visium Spatial Gene Expression slide, which contains spatially unique capture oligos. Following staining and imaging, histological sections were hybridized using a comprehensive mouse whole transcriptome probe set mapping to ~20 000 genes. Following probe ligation and rinsing, specifically hybridized probes were then released from the tissue through permeabilization and captured by Visium slide oligos. Subsequent sequencing, alignment, and registration to the H&E-stained image resulted in gene expression information within its original 2D position within the bone. Following exclusion of surrounding soft tissue, as well as cartilage from the growth plate, quality controls were applied to assess the transcript recovery efficiency (Fig. 1a). To determine tissue-specific gene changes, we manually segmented spatial samples into cortical bone (orange), trabecular bone (blue), and bone marrow (magenta) (Fig. 1b). Combined, our assessment showed an average of 172 ± 154 (cortical), 194 ± 96 (trabecular), and 233 ± 131 (marrow) unique genes per spot for a combined 13 115 (cortical), 9 605 (trabecular), and 17 524 (marrow) total unique genes represented within each of the morphologically unique regions. Notably, spatial spots were assigned to the tissue most represented within each spot and, as a result, may partially contain cells from neighboring tissue regions (i.e., spots designated as “cortical bone” overlay the cortical bone by >50% but may also partially overlap with neighboring bone marrow). Gene expression profiles were used to confirm the successful spatial delineation of captured transcripts (Fig. 1c). Both cortical and trabecular bone showed high levels of mature osteogenic transcripts (*Col1a1, Bglap*), with additional periosteal (*Postn*) and osteoprogenitor (*Sp7, Runx2*) genes enriched within the cortical and trabecular bone, respectively. In contrast, hematopoietic (*Ptprc*, encoding CD45), erythropoietic (*Hba-a2*), and proliferative markers were heavily enriched within the bone marrow (Fig. 1c). These data show for the first time the technical feasibility of conducting spatial transcriptomics within fully mineralized adult long bone tissue.

### Computational deconvolution of spatial spots using scRNAseq

**Fig. 2:**
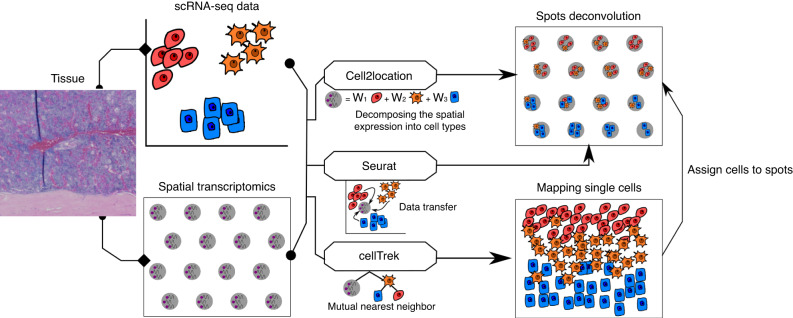
Spatial spot deconvolution using scRNAseq. Overview of the deconvolution of spatial spots. First, scRNAseq data and spatial data are collected from the same or similar tissue. Second, scRNAseq data and spatial transcriptomics are then fed to three different deconvolution algorithms: Cell2Location, Seurat, and CellTrek. Cell2Location uses a Bayesian model to decompose the spatial expression count matrix into cell type signatures. Seurat employs bulk gene expression deconvolution based on a single-cell reference. CellTrek maps single cells to spatial locations. Finally, prediction results of the cell type abundance at each spatial spot are generated from the three algorithms

**Fig. 3:**
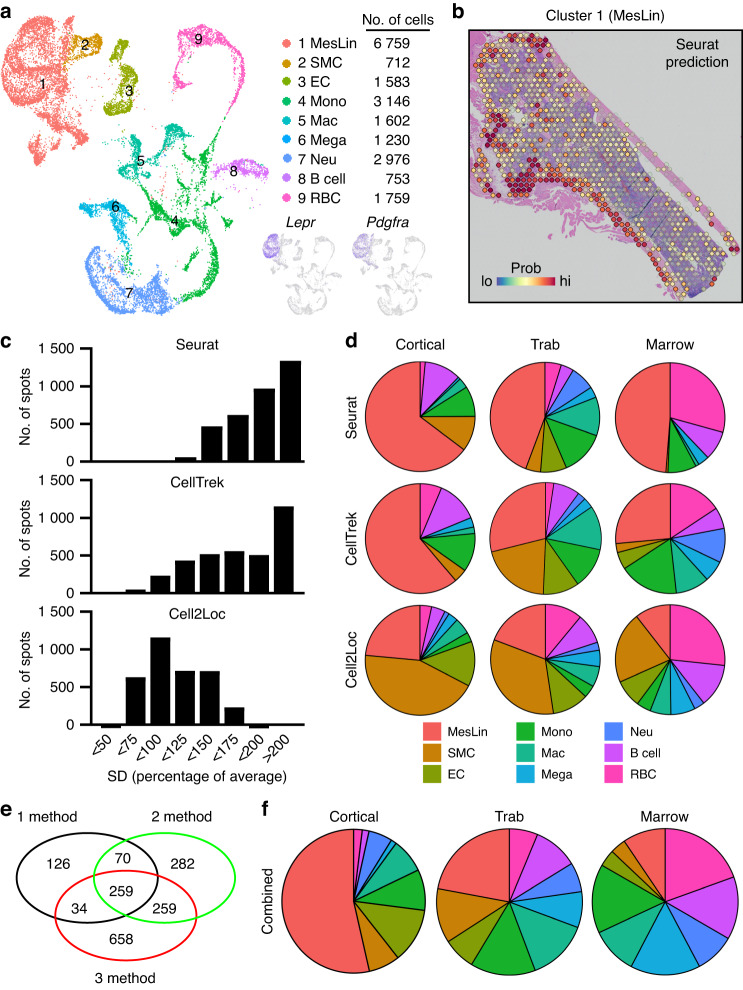
Compositional deconvolution of spatial spots using scRNAseq. **a** UMAP of bone marrow scRNAseq datasets. **b** Probability that spatial spots contain MesLin cells. **c** Standard deviation (SD) of predictive values for each cell cluster within each spatial spot. Higher values denote greater variation in predictive values and a greater discernment of cell types within each spatial spot. **d** Pie chart showing the probability of each indicated cell type being assigned to each spatial region using the three indicated predictive packages. **e** Overlap between prediction methods (Seurat, CellTrek, and Cell2Location) of spatial spots proposed to contain MesLin cells. **f** Pie chart showing the probability of each indicated cell type being assigned to each spatial region using the combined predictive pipeline

The high complexity of the bone marrow in terms of the diversity of cell types present in close proximity of each other, in combination with the limited spatial resolution of spatial transcriptomics, make it challenging to assign spatial gene expression profiles to individual cell types. To overcome this, we made use of scRNAseq to computationally deconvolve spatial spots into their cellular constituents. Deconvolution of spatial spots was conducted using scRNAseq datasets and the packages Seurat, CellTrek, and Cell2Location (Fig. 2). Seurat relies on data transfer of uniquely expressed genes between cellular clusters, CellTrek uses a mutual nearest neighbor-based approach to map individual cells to spatial data, and Cell2Location uses a decomposition-based approach. For our studies, multiple scRNAseq datasets were combined from bone- and marrow-derived cells^5,7^ to generate a single object comprising all major cell types of the bone marrow (Figs. 3a and S1). Seurat, CellTrek, and Cell2Location each provided a probability in which an scRNAseq cluster of interest is present within each spatial spot (Fig. 3b). Predictive modeling was conducted for all clusters and mapped to all regions of our spatial data. The discerning power of each predictive model was then determined by calculating the standard deviation in predictive scores within a single spatial spot across all clusters (larger values denote an increased ability to distinguish the enrichment of an individual cell type above random mixing) (Fig. 3c). Next, predictive values were interrogated in terms of their cellular placement within the different spatial compartments (Fig. 3d). These results suggest that each of the predictive models possesses positive and negative attributes. Seurat showed a relatively high abundance of mesenchymal lineage (MesLin) cells within the cortical (65%) and trabecular (44%) bone, as well as within the marrow (49%). Conversely, Cell2Location showed a high abundance of hematopoietic cells within the marrow (63%) but a disproportional abundance of smooth muscle cells (SMCs) within both cortical (44% vs. 11% and 4% in Seurat and CellTrek, respectively) and trabecular (33% vs. 4% and 21% in Seurat and CellTrek, respectively) bone regions. CellTrek appeared to perform more moderately in both assigning MesLin cells to bone compartments and hematopoietic cells to the marrow compared to Seurat and Cell2Location (Fig. 3d). To overcome these limitations within each predictive package, we created a combined predictive pipeline. All spatial spots within the upper 2 quartiles of predictive values were selected for each of the 3 predictive models and compared to determine the level of overlap between each method (Fig. 3e). Spots in the upper 2 quartile predictive values in at least 2 separate methods were defined as positive for each cell type. The distribution of cells using our combined predictive pipeline showed strong mapping of MesLin cells to cortical bone (53%) and hematopoietic cells to the marrow compartment (83%) (Fig. 3f), consistent with the known cellular composition of these tissues. The presence of hematopoietic cells within the cortical (27%) and trabecular (59%) compartments likely reflects the fact that spatial spots designated as cortical or trabecular bone partially overlap with the neighboring marrow.

**Fig. 4:**
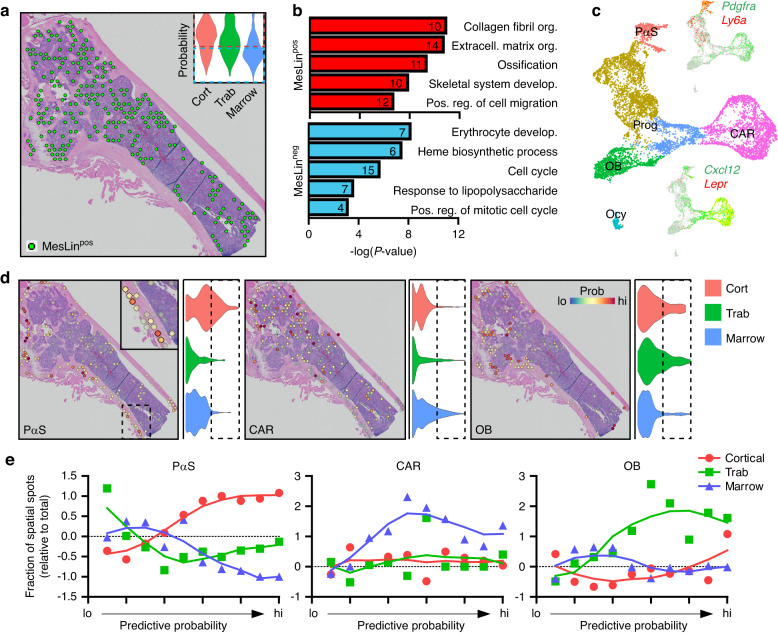
Proposed SSPC subtypes localize to spatially distinct skeletal regions. **a** Spatial spots identified as containing MesLin cells within the marrow. The insert shows the probability of MesLin cells localizing to each of the spatial regions. Dotted boxes denote the upper (red) and lower (blue) 2 quartiles. **b** Pathway analysis of marrow DEGs enriched in MesLin^+^ and MesLin^−^ spatial spots. **c** UMAP of subclustered MesLin cells. **d** Predictive modeling of the indicated single-cell cluster onto spatial spots. **e** Predictive modeling quantification of the indicated MesLin subcluster onto segmented spatial data

With this analytical pipeline, MesLin cells showed increased predictive probabilities within the cortical and, to a lesser extent, trabecular spatial spots (Fig. 4a, insert), likely corresponding to the high enrichment of osteoblasts and osteocytes within the bone. For confirmation of the use of these selection criteria, MesLin^+^ spatial spots were isolated from the marrow (Fig. 4a), and differentially expressed genes were calculated on MesLin^+^ marrow spots relative to MesLin^−^ marrow spots. Pathway analysis of DEGs showed enrichment in terms linked to matrix production/organization, as well as skeletal development and ossification, within MesLin^+^ spots, while MesLin^−^ spots were highly enriched for erythropoiesis, immune cells, and cell proliferation (Fig. 4b). To further refine this spatial localization of scRNAseq clusters, we next reanalyzed and subclustered MesLin cells (Fig. 4c). The expression pattern of marker genes (Fig. 4c and Table S1) revealed mature osteoblasts (OBs) and early osteocytes (Ocys), along with two previously proposed, heterogeneous SSPC populations, PαS cells, defined here as expressing high levels of *Pdgfra* and *Ly6a* (encoding SCA1) (PαS), and CXCL12-abundant reticular (CAR) cells, demarcated here by the expression of *Cxcl12* and *Lepr*. To characterize the spatial distribution of these SSPC clusters, we applied a similar predictive modeling workflow as described above, with MesLin^+^ spatial spots further divided into these two proposed SSPC cell subtypes, along with mature osteoblasts to serve as a control (Fig. 4d). Quantitative analyses of subtype distribution suggest that PαS cells were highly enriched within the cortical bone, especially along the outer cortical surface consistent with the periosteum (Fig. 4d, e). In contrast, CAR cells were found primarily within the bone marrow, consistent with bone marrow SSPCs. Osteoblasts were associated primarily with trabecular spatial spots and to a much lesser extent within cortical spatial spots, consistent with the high bone remodeling frequently observed within trabecular compartments. Our combined predictive pipeline approach demonstrates the collective power of spatial transcriptomics and scRNAseq to overcome limitations in spatial resolution and spatially localize PαS and CAR cells to the periosteum and marrow, respectively.

### Defining the cellular components of the marrow SSPC niche

**Fig. 5:**
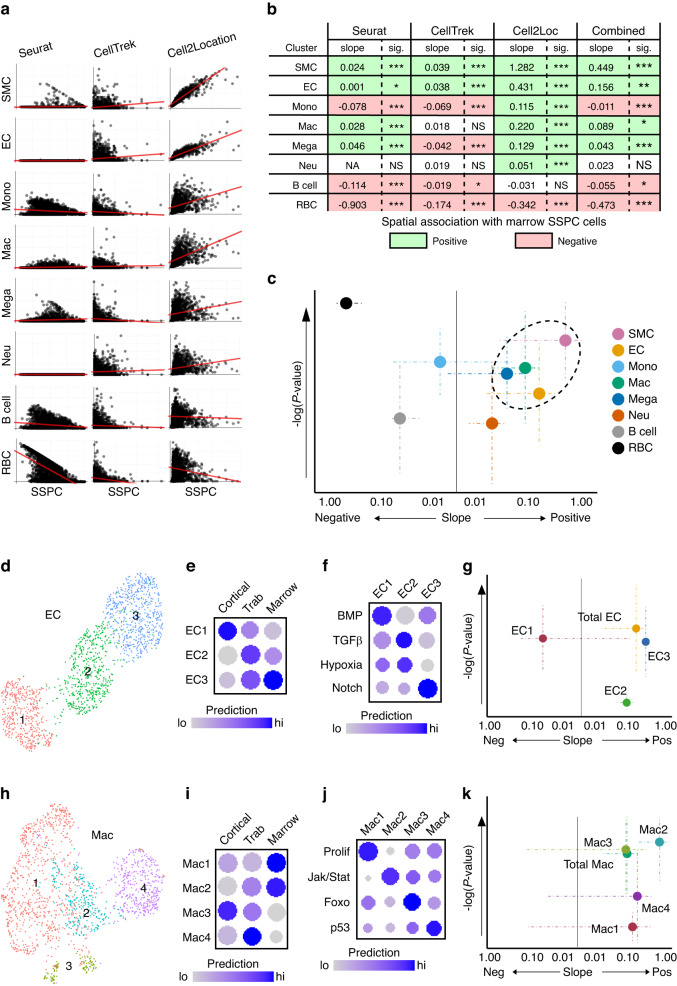
Identification of niche-resident cells. **a** Probability correlations indicating the likelihood that cell types are present within marrow *Cxcl12*^*+*^*Lepr*^*+*^ SSPC spatial spots. **b** Statistical analyses showing the likelihood that the indicated cell is present within the stem cell niche. Green values indicate significant positive enrichment; red values indicate significant negative enrichment. **c** Bubble plot showing cell types predicted to be within the niche. Cell types within the dotted boundary were significantly enriched. **d** UMAP of subclustered ECs. **e** Dot plot showing predictive values of where each EC subcluster maps to each spatial transcriptomic region. **f** Dot plot showing modular scoring of pathway activation within each EC subcluster. **g** Bubble plot showing EC subclusters predicted to be within the SSPC niche. **h** UMAP of subclustered Macs. **i** Dot plot showing predictive values of where each Mac subcluster maps to each spatial transcriptomic region. **j** Dot plot showing modular scoring of pathway activation within each Mac subcluster. **k** Bubble plot showing Mac subclusters predicted to be within the SSPC niche

Having computationally derived the spatial locations of this heterogeneous *Cxcl12*^*+*^*Lepr*^*+*^ SSPC population within the bone marrow, we next investigated the other cellular components present within the bone marrow SSPC niche. We combined our predictive pipeline data for each cell cluster (Fig. 3f). Next, correlative analyses were conducted to determine the probability that SSPCs and each indicated cell type are present within the same spatial spots (i.e., which cell types are frequently found to be present within the same spatial location as SSPCs) (Fig. 5a). Correlations using all three predictive modeling packages (Seurat, CellTrek, Cell2Location) were used to quantify the slope of the spatial correlations between SSPCs and the cell types of interest (in log scale) and the significance of this correlation (Fig. 5b). These results suggest that *Cxcl12*^*+*^*Lepr*^*+*^ SSPCs within the bone marrow are most frequently spatially associated with smooth muscle cells, endothelial cells, macrophages, and, to a lesser extent, megakaryocytes. Conversely, SSPCs show a strong, negative spatial correlation with red blood cells (Fig. 5c). These results recapitulate previous works in the stem cell field,^13^ validating this approach to assess the cellular composition of the niche using spatial transcriptomics and our deconvolution pipeline.

To further refine the cellular constituents present within the bone marrow *Cxcl12*^*+*^*Lepr*^*+*^ SSPC niche, we subclustered and reanalyzed endothelial cell (Fig. 5d–g) and macrophage (Fig. 5h–k) single-cell populations for their association with SSPC-containing spatial spots. Endothelial cells were subclustered into 3 transcriptionally unique subpopulations (EC1-3) (Fig. 5d). EC1 was enriched for marker genes for sinusoidal vessels, while EC3 was enriched for arteriole markers^4^ (Fig. S3A). EC2 showed moderate expression for each vessel type but was enriched for angiogenic tip cells vs. stalk endothelial cells^25^ (Fig. S3B, C). These 3 endothelial cell subclusters exhibited differential mapping to our spatial data, with EC1 most closely associated with cortical bone surfaces, EC2 associated primarily with trabecular bone, and EC3 associated with the bone marrow and, to a lesser extent, trabecular bone (Fig. 5e). Pathway analysis of subcluster DEGs (Table S2) showed enriched activation of BMP signaling in EC1, TGFβ and hypoxia-related signaling in EC2, and Notch signaling in EC3 (Fig. 5f). Calculation of the correlation slopes revealed that EC3 was significantly and positively correlated with SSPC-containing bone marrow spatial spots, while the EC1 and EC2 subpopulations showed a strong negative and weak positive correlation, respectively (Figs. 5g and S2A). Similar subcluster analyses were conducted on macrophages (Mac1–4) (Fig. 5h), with spatial mapping showing distinct areas of enrichment for each of the 4 macrophage subclusters (Fig. 5i). Pathway analyses from subcluster DEGs (Table S3) showed unique enrichment in several pathways linked to cellular function and inflammatory status (Fig. 5j). Calculating the correlation slopes revealed that Mac2 was significantly and positively correlated with SSPC-containing bone marrow spatial spots (Figs. 5k and S2B). Mac3 cells showed a positive correlation with SSPC-containing spots similar to the total macrophage population but were not significantly enriched due to high variance in the correlative slopes between prediction methods (combined *P* value = 0.109 2), while both Mac1 and Mac4 associations were found to not be significant (Figs. 5k and S2B). These data demonstrate the feasibility of using single-cell prediction methods combined with spatial transcriptomics to identify heterogeneous populations of cell types frequently present within the stem cell niche. Furthermore, these results demonstrate that these cell types can be segmented by gene expression profiles and spatially subdivided to quantify enrichment within the SSPC niche.

### Dissecting cell‒cell interactions within the SSPC niche

**Fig. 6:**
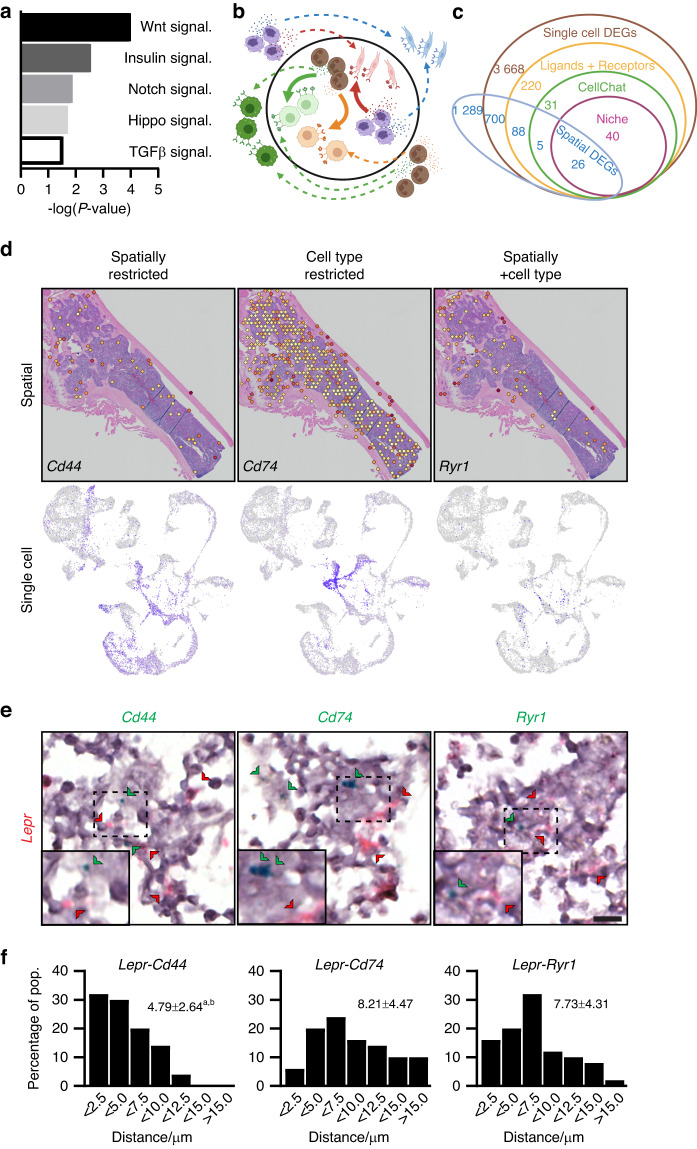
Signaling within the stem cell niche using spatial transcriptomics. **a** Pathway analysis of DEGs enriched with niche spatial spots relative to other spatial spots within the bone marrow. **b** Schematic of cell‒cell interactions. While scRNAseq can be used to predict ligand/receptor pairs between cell types, the addition of spatial information can distinguish biologically relevant interactions occurring between cells with close proximity (within circle, solid arrows) from those occurring between distant cells unlikely to occur in vivo (outside of circle, dotted arrows). **c** Predicted cell‒cell interactions using single-cell spatial transcriptomics. Genes were defined as showing enriched expression within select cell types (brown), encoding known receptors or ligands (yellow), predicted to communicate using *CellChat* (green), and being expressed by those cell types present within the stem cell niche (magenta). Genes were overlaid with spatial DEGs (blue). **d** Sample genes identified from cell‒cell interaction found to be spatially restricted to the niche but expressed by numerous different cell types (*Cd44*), genes expressed by only select cell type but present throughout the marrow (*Cd74*), and genes restricted both spatially and by cell type (*Ryr1*). **e** RNAscope of bone marrow showing *Lepr*-expressing SSPCs (red) and *Cd44* (left), *Cd74* (middle) and *Ryr1* (right) (green). Bar, 10 µm. **f** Distribution of distances between the indicated niche cell markers and *Lepr*^+^ SSPCs. Numbers above the graph represent the average±SD of the distance between cells. *n* = 50 cells per marker. ^a^*P* < 0.001 vs. *Cd74*, ^b^*P* < 0.001 vs. *Ryr1*

Having established the cell types frequently present within the SSPC niche, we next sought to determine the signaling mechanisms occurring within the stem cell microenvironment. Differential gene expression analysis was conducted on SSPC^+^ spatial spots within the marrow relative to other marrow spots using each of the 3 methods of single-cell predictions (Table S4) or our combined method approach (Table S5). Pathway analysis of genes enriched within SSPC-containing marrow spots revealed enriched expression in several well-established morphogenetic pathways, including WNT, Notch, and TGFβ signaling (Fig. 6a). Next, we sought to use ligand‒receptor interactions to understand how these and other signaling cascades are regulated within the SSPC niche. Single-cell analysis packages have been previously developed to identify potential cell‒cell interaction mechanisms.^26–28^ However, they lack the spatial information to determine whether the proposed cell types both exist within a sufficiently close proximity and express the proposed ligand and receptor within this region. As such, it is challenging to distinguish computationally predicted (Fig. 6b, dotted arrows) from biologically relevant (Fig. 6b, solid arrows) signaling mechanisms. To overcome these limitations, we combined single-cell data with our spatial data to refine our list of total spatial and single-cell cluster DEGs into those genes that expressed known ligands or receptors, which were predicted to interact between cell types by *CellChat*, were mechanisms of cell‒cell interaction between the cell types predicted to be present within the SSPC niche and finally showed enriched expression within our predicted SSPC^+^ niche spatial spots (Fig. 6c and Table S6). Although unable to distinguish finer spatial organization, this combinatorial approach of scRNAseq and spatial transcriptomics can identify genes enriched within a 55 µm area (size of each spatial spot) around the predicted SSPC location. Probing of these different categories of overlapping DEGs allowed us to identify (i) niche signaling factors spatially restricted to the niche but showing widespread expression across multiple cell types (spatially restricted), (ii) genes that were enriched within cell types but not spatially restricted to the niche (cell type restricted), and (iii) genes that were found to show both cell type and spatially restricted expression (spatial and cell type restricted) (Fig. 6d). Similar results were obtained using RNAscope for *Cd44-*, *Cd74-*, and *Ryr1*-expressing cells surrounding *Lepr*^+^ SSPCs (Fig. 6e). Quantification of these in vivo results shows that while *Cd44*^+^ and, to a lesser extent, *Ryr1*^+^ cells are spatially restricted to within a few µm of *Lepr*^+^ SSPCs, *Cd74*^+^ cells were more ubiquitously dispersed (Fig. 6f). These data suggest that combining spatial and single-cell analyses can localize potential ligand‒receptor cell‒cell interactions predicted by scRNAseq algorithms within SSPC niche signaling in an unbiased manner to reveal new components of niche biology.

### Signal gradients establish domains within the bone marrow

**Fig. 7:**
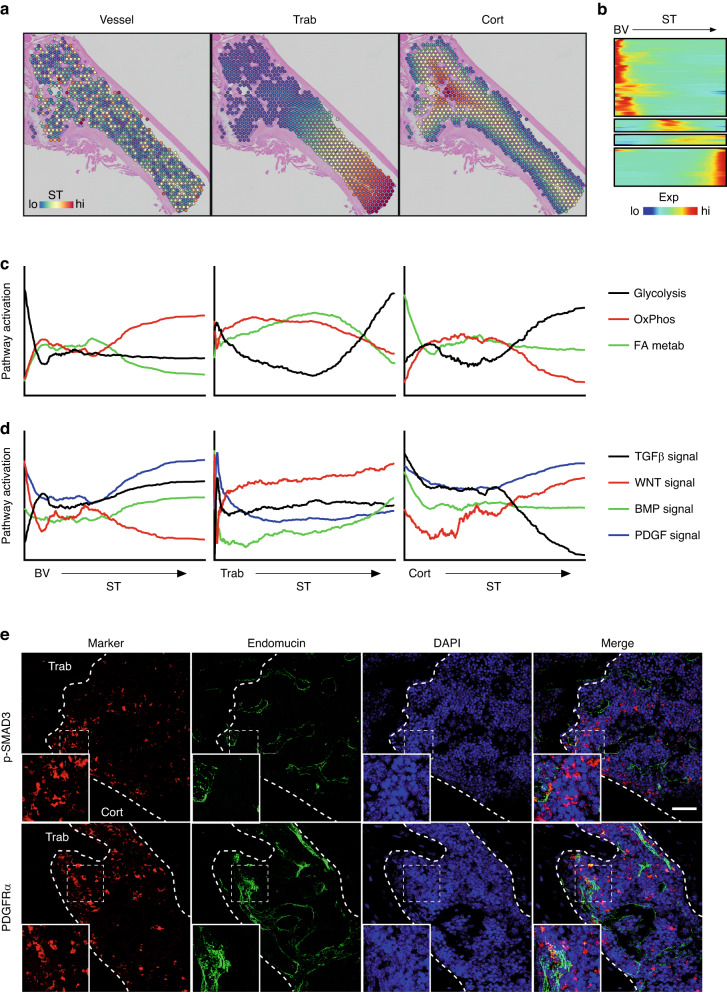
Spatial-time analysis reveals gradients of signals originating from the blood vessels. **a** Spatial distance calculations showing the minimal distance of each marrow spatial spot to the nearest vessel (left), trabecular bone surface (middle), and cortical bone surface (right). **b** Identification of genes that are differentially regulated across the marrow as a function of their distance to the nearest blood vessel. **c** Module scoring showing activation of the indicated metabolic pathways within marrow spatial spots relative to their distance to the nearest blood vessel (left), trabecular (middle), or cortical (right) surface. **d** Module scoring showing activation of the indicated morphogenetic pathways within marrow spatial spots relative to their distance to the nearest blood vessel (left), trabecular (middle), or cortical (right) surface. **e** Immunofluorescence staining of TGFβ activation (p-SMAD3) and PDGFRα in mouse long bone. Blood vessels are denoted by endomucin (green). Dotted lines denote the bone boundary. Bar, 100 µm

In addition to local signaling axes present within the SSPC niche, we further hypothesized that broader signaling gradients exist within the bone marrow that establish domains within the marrow cavity. These microdomains would be the result of secreted ligands or the availability of nutrients and oxygen and act in a coordinated fashion, likely affecting all cells based on their proximity to major tissue areas such as the bone surface or blood vessels. To assess these gradients in an unbiased fashion, we conducted spatial-time analyses on marrow spatial spots (Fig. 7). This technique utilizes a manually designated reference line and then measures the distance between each spatial spot and the nearest point along this reference surface.^24^ First, spatial spots were aligned based on their relative proximity to the nearest blood vessel, trabecular bone, or cortical bone surface (Fig. 7a). Genes whose expression fluctuated as a function of their proximity to their reference surface were then identified (Fig. 7b). Pathway analysis was then used to identify major regulatory networks altered relative to their distance from the reference tissue.

To observe overall pathway activation across SpatialTime as a function of the distance from the nearest vessel, trabecular, or cortical bone surface, we conducted module scoring, which calculates the average expression of a gene list curated from known KEGG pathways relative to background. Scoring for genes linked to bioenergetics, we observed high levels of glycolytic gene expression immediately adjacent to blood vessels, immediately adjacent and distant from the trabecular surface, and distant from the cortical bone surface (Fig. 7c). In contrast, genes linked to oxidative phosphorylation (OxPhos) were predominantly found within spatial marrow spots distant from blood vessels and intermediate from both trabecular and cortical bone surfaces. Finally, fatty acid (FA) metabolism was found to be predominantly associated with the cortical bone surface and at intermediate distances from trabecular bone surfaces (Fig. 7c). In addition to metabolic regulation, pathway analysis identified changes in gradients of signal activation associated with major morphogenetic pathways (Fig. 7d). Platelet-derived growth factor (PDGF) signaling was found to be highly active both adjacent to and distant from blood vessels, as well as adjacent to trabecular bone surfaces. In contrast, WNT signaling was high immediately adjacent to blood vessels and distant from both trabecular and bone surfaces, while WNT signaling appeared conversely low distant to vessels and adjacent to trabecular and cortical bone surfaces (Fig. 7d). Both bone morphogenetic protein (BMP) and transforming growth factor beta (TGFβ) signaling were preferentially high near trabecular and cortical surfaces and declined at more distant marrow spots (Fig. 7d). Histological validation confirmed the preferential activation of TGFβ signaling (denoted by p-SMAD3 staining) near cortical and trabecular surfaces, as well as preferential staining for PDGF receptor alpha (PDGFRα) near trabecular vascular surfaces (Fig. 7e). Overall, these unbiased analyses identify microdomains of signaling gradients, cellular function, and metabolism in response to the distance from blood vessels and bone surfaces.

## Discussion

In this study, we used an analytical pipeline to probe signaling within the stromal cell niche within the bone. ScRNAseq suffers from a lack of spatial context, while spatial transcriptomics lacks the resolution to distinguish individual cells. Our approach overcomes the spatial limitation of scRNAseq and resolution limitation of spatial transcriptomics for the first time in the long bones of adult mice through the use of predictive modeling and deconvolution. Here, we used this approach to show the spatial location, cellular composition, and cell‒cell communication network of previously proposed stromal cell populations in the niche, placing them in the context of regulatory microdomains within the marrow.

While scRNAseq analyses have substantially advanced our understanding of stromal cells and their commitment to cell-specific lineages within the bone, some disparity remains in terms of the heterogeneity of proposed stromal cell populations. Prior immunohistochemical and in situ studies have identified several SSPC markers, including PDGFRα/SCA1,^10^ CXCL12,^9^ and LEPR.^10^ However, further refinement using scRNAseq analysis suggests the possibility of two distinct SSPC populations.^5,7,12^ To attempt to resolve these findings, we used scRNAseq and spatial transcriptomics data to create an analytical predictive pipeline using the previously generated *R* packages Seurat,^29^ CellTrek,^30^ and Cell2Location.^31^ Although each proposed population is composed of a heterogeneous set of stem/progenitor/stromal cells, for simplicity, this study used this analytical pipeline to probe general populations of SSPCs expressing high levels of *Pdgfra* and *Ly6a* (PαS) or *Cxcl12* and *Lepr* (CAR). Using our predictive pipeline, we exploited the strengths of each analytical approach to create a predictive workflow for the location of each cell cluster within complex and heterogeneous spatial data. Our spatial predictive mapping suggests that CAR cells are heavily enriched within the bone marrow, consistent with previously published results.^32–34^ In contrast, while some PαS cells were found within the bone marrow, these cells were heavily enriched within the cortical tissue primarily along the outer periosteal surface. This finding is consistent with more recent studies identifying PDGFRα and SCA1 as markers of periosteal stem cells important for fracture healing.^35–37^ These findings suggest that either PαS cells present within the bone marrow show significant overlap in gene expression with stem cells within the outer periosteum or that periosteal cells may have been inadvertently captured during enzymatic digestion to generate bone marrow single-cell preparations.

SSPCs rely on the regulatory function of other cell types within the stem cell niche to be maintained in an undifferentiated state. To date, several cell‒cell communication prediction packages have been developed to infer ligand‒receptor-based interactions. However, one striking limitation of these methods remains the lack of spatial information. As a result, while these communication packages can predict potential mechanisms through which crosstalk may occur, they lack the ability to determine if the proposed crosstalk, while theoretically possible, is biologically feasible (i.e., do the cells proposed to communicate exist within spatial confines that would make direct cell‒cell communication possible while also expressing the necessary genes for interaction within this spatial context). To overcome this limitation, we made use of the fact that spatial transcriptomics is not at a single-cell resolution and instead considered each spatial spot at the “niche” level. We determined the probability that other cell types of the bone marrow were present within the same spatial spot as our previously identified SSPC populations. Consistent with previously published results, our data indicate that smooth muscle cells, endothelial cells, macrophages, and, to a lesser extent, megakaryocytes comprise the SSPC niche. Reanalysis of both endothelial cells and macrophages identified cellular subtypes enriched within the niche. Transcriptional analyses of the endothelial and macrophage subtypes present within the niche revealed enriched activation of Notch and Jak/Stat signaling, respectively. Previous studies have shown the critical role of endothelial Notch signaling in regulating hematopoiesis,^12^ as well as in regulating SSPC proliferation and differentiation.^38–40^ Conversely, other studies have shown that mesenchymal stromal cells induce M2 polarization within macrophages coinciding with elevated levels of Jak/Stat signaling.^41,42^ Notably, due to the spatial resolution (55 µm), our signaling analyses cannot distinguish cells in immediate contact with SSPCs from those present within the same spatial spot but still upward of several cell lengths removed from the SSPC itself. While this method showed improvements compared to analyses relying on scRNAseq alone, pathways such as Notch, which require direct cell‒cell interaction, may still be falsely predicted. Combining spatial transcriptomics, scRNAseq, and our spatial deconvolution pipeline, our results demonstrate the ability to dissect cellular composition and communication within the niche in an unbiased fashion.

While the niche has been extensively investigated, one concept that is well known, yet difficult to assess, is the formation of microdomains within the bone marrow regulated by the release of growth factors from various tissues. Osteoblasts and osteoclasts along bone surfaces, chondrocytes at the terminus of the growth plate, and vascular endothelial and smooth muscle cells have all been shown to regulate the local environment. However, assessing these signaling gradients in an unbiased fashion has proven difficult with existing techniques. Our analyses revealed unique expression of transcripts related to metabolism and major morphogenetic pathways. Despite the presumably high availability of oxygen near blood vessels, we observed low levels of oxidative phosphorylation and high levels of glycolysis within spots immediately adjacent to vascular tissue. These results are consistent with previous data that suggest that endothelial cells,^43^ smooth muscle cells,^44^ and skeletal stem cells^45^ all favor glycolysis as an energy production mechanism to minimize ROS production^43,46^ and overall gene activation.^47,48^ Additionally, these results showed elevated levels of transcripts associated with FA metabolism near the cortical bone surface. This finding is consistent with in vitro studies that show increased FA metabolism in mature osteoblasts^49^ and indicate the importance of FA metabolism in normal bone accrual^50^ and the response to anabolic stimuli.^51^ Similarly, previous findings of elevated WNT^52,53^ and PDGF^54,55^ signaling near vessels as well as high activation of PDGF^6,56^ and BMP^57,58^ near bone surfaces were reflected in this analysis. Our findings further add indications of the potential range of these coordinate gradients, with, for example, WNT signaling showing a more restrictive window of activation surrounding blood vessels than that observed by PDGF signaling.

Several potential limitations should be considered in this analytical pipeline. The unique anatomy of bone, with a mineralized outer cortical shell, can delay the diffusion of reagents to the internal marrow and necessitate decalcification, ultimately delaying sample processing. These factors may contribute to the relatively low number of transcripts retrieved compared to those of previous works in soft tissue. While previous studies using scRNAseq of bone-derived cells or spatial transcriptomics of soft tissue typically yield several thousand transcripts per cell/spot, our results show a greatly reduced efficiency. As such, limitations in the extent and types of analyses possible should be carefully considered. These limitations were overcome, to some extent, by (i) more broadly selecting our cell clusters of interest for spatial mapping, (ii) investigating expression patterns of groups of genes linked to a common pathway, (iii) relying on a combination of predictive packages, and (iv) using techniques such as SpatialTime to allow the merging of multiple samples into a single analysis. Optimization of this sample preparation pipeline may yield greater transcript recovery, increasing the accuracy of downstream analyses and permitting more sophisticated investigations in the future. While we speculate that our findings will be similar across mouse strains, our spatial analyses are specific to C57BL/6 mice. Additional studies are needed to determine global versus strain-specific findings.

Overall, this study makes use of predictive modeling, combining the unbiased transcriptional profile and in vivo spatial context of spatial transcriptomics with the cellular resolution of scRNAseq. These studies reveal the cellular and signaling components of the stem cell niche, as well as the gradients of signals established by various anatomical structures within the bone. These analyses are highly supportive of intensive studies published over the past several decades and suggest that this analytical pipeline could serve as a method to unbiasedly assess changes in several aspects of stem cell and bone biology in response to perturbation or therapeutic intervention.

## Materials and methods

### Spatial transcriptomics of bone

Femurs from 2 separate mice were harvested from 10-week-old C57BL/6 mice (Charles River), bisected, and immediately fixed in 10% buffered formalin overnight at 4 °C. The samples were decalcified in 0.5 mol·L^−1^pH 8 EDTA for 2 weeks on a shaker, with a fresh reagent change every 1–2 days. The samples were then processed for paraffin embedding. All processing was conducted with minimal delay between steps. Two 5 µm longitudinal sections through the marrow were collected per block from two different FFPE blocks (for a total of 4 tissue sections) and placed onto the Visium Spatial Gene Expression Slide (10 x Genomics). Spatial transcriptomic libraries were generated using the Visium Spatial Gene Expression for FFPE Kit according to the manufacturer’s instructions (10 x Genomics, CG000407, Rev D). Briefly, the slide was subjected to H&E staining and imaged at × 20 magnification using a NanoZoomer S60v2MD (Hamamatsu). Tissue sections were decrosslinked and probe-hybridized (Visium Mouse Transcriptome Probe Set v1.0), targeting 20 551 mouse genes. Specifically, hybridized and ligated probes were then released from the tissue through permeabilization and captured by Visium Slide oligos. Barcoded ligation products were then subjected to amplification and indexing. Libraries were pooled and subjected to 2 × 96 bp pair-ended sequencing with a sequencing depth of >100 M reads per sample on a NovaSeq instrument (Illumina). Demultiplexing and sequence alignment, along with registration to H&E-stained images, was carried out using the *SpaceRanger* pipeline.

### Computational analyses of spatial and single cells

Analyses of both spatial and scRNAseq data were carried out in the *R* package *Seurat*.^29^ For spatial data, histologically unique regions of the bone were manually segmented, and differential gene expression was carried out using default parameters. For whole bone marrow single-cell analyses, datasets from GSE145477 (GSM4318799)^7^ and GSE128423 (GSM3674243, GSM3674244, GSM3674245, and GSM3674246)^5^ were merged into a single object to represent all major types of cells present within the bone marrow. For analysis of mesenchymal lineage cells, datasets from GSE145477 (GSM4318799, GSM4318800, and GSM4318801)^7^ were reanalyzed. Pathway analysis of DEGs was conducted using the Database for Annotation, Visualization and Integrated Discovery (DAVID).^59^ The *AddModuleScore* function was used to assess levels of overall pathway activation using established gene lists curated from KEGG pathways (Table S7). For determination of the potential interactions between different cell clusters, we performed cell‒cell communication analysis using the *R* package *CellChat*.^60^

### Deconvolution of spatial spots using scRNAseq

To deconvolve spatial spots into single cells, we separately utilized the predictive tools Seurat, Cell2Location, and CellTrek to estimate the abundance/proportion of different scRNAseq-derived cell types in each spatial spot. Seurat first identified transferable anchors for each cell type of the scRNAseq data and then extracted the expression matrices of these anchors from scRNAseq data and spatial data, which were subsequently used as input in the deconvolution algorithm SCDC to acquire predicted proportions of each cell type for each spatial spot. Cell2Location is a Bayesian model that predicts the abundance of cell types at each spatial location. It builds linear regressions to describe the relationship between gene expression at a spatial location and gene expression of each cell type while also weighing information shared across spatial locations to obtain the regression weights of each cell type, which are interpreted as the cell type abundance. Unlike the other two methods that directly predict the abundance of cell types, CellTrek is a single-cell mapping tool. ScRNAseq and spatial data were first coembedded, and then, a random forest distance between single cells and spatial spots was computed. Finally, using mutual nearest neighbor calculations, spatial coordinates were transferred to single cells. Based on the spatial coordinates of single cells, we assigned each single cell to its nearest spot before calculating the cell abundance and proportion. To determine the cellular components of the niche, we conducted linear correlations on each marrow spot for SSPCs and the indicated single-cell cluster using predictive values derived from each of the three predictive packages. Significance was assessed by the probability that the correlative slope was equal to 0. The slope and *P* value were averaged to obtain combined values.

### SpatialTime analysis

SpatialTime analyses were conducted according to previously published methods.^23,24^ Briefly, blood vessel, trabecular, and cortical surfaces were manually contoured. Next, distances were calculated between each spatial spot within the marrow and each pixel along the manually drawn surface contours, with the minimum distance selected. Finally, distances were normalized to values between 0 (immediately adjacent to contoured surface) and 1 (most distant from surface). For analysis of genes differentially expressed across each of these spatial-time axes, marrow spatial spots were analyzed by the *R* package *Monocle* using the *differentialGeneTest* function, replacing pseudotime values with self-calculated spatial-time values. For visualization of changes in cellular pathways, module scores were calculated across SpatialTime values, and a smoothed curve was generated by averaging neighboring spot values.

### Immunofluorescence and RNAscope

For immunofluorescence, long bones from 12-week-old mice were isolated and fixed in 4% PFA overnight. Bones were then decalcified in Decalcifier I (3800440, Leica) for 4 days. Bones were cryoprotected in 20% sucrose and 2% polyvinyl pyrrolidine (PVP) and then embedded in 20% sucrose, 2% PVP, and 8% gelatin. Sections were cut at a thickness of 100 µm and stained using primary antibodies against endomucin (Sc-659495, Santa Cruz), PDGFRα (AF1062, R&D Bioscience), or p-SMAD3 (ab52903, Abcam), followed by secondary antibodies (Alexa Fluor 488 donkey anti-rat, Alexa Fluor 647 donkey anti-goat, Alexa Fluor 555 donkey anti-rabbit, Invitrogen). Images were acquired using a Leica DMI8 confocal microscope. Images are representative of three individual samples taken from three separate mice.

To detect single mRNA molecules, we performed FFPE-fixed RNAscope on sections from control C57BL/6 mice (Charles River). Positive control probes (the *Mus musculus* duplex probes *Ppib*, green channel, and *Polr2a*, red channel, and the single channel *Ubc* control to test for high expression in bone) and one negative control probe (*Escherichia coli DapB*) were used. Following protocol optimization, sections were assessed for *Lepr* (C1) and either *Cd44* (C2), *Cd74* (C2), or *Ryr1* (C2). In situ hybridization was performed according to the protocol of the RNAscope 2.5 Duplex Detection Kit (Chromogenic, Cat. No. 322500). Briefly, sections from FFPE mouse tibias and an RNAscope Control Slide (Mouse 3T3 cell pellet, Cat. No. 310023) were baked at 60 °C for 1 h before deparaffinization and dehydration according to the protocol. The slides were then incubated with H_2_O_2_ and permeabilized before air drying. C2 probes were diluted in C1 probes at a 1:50 ratio and incubated on the slides for 2 h at 40 °C and amplified according to the protocol. Positive *Ppib/Polr2a* duplex probes, *DapB* negative probe, and *Ubc* control probe were incubated separately on three individual tibia samples. The RNAscope Control slide received the duplex control probes. Prior to coverslipping, slides were stained with hematoxylin. Images are representative of three individual samples. For quantification of RNAscope, 50 *Cd44*^+^, *Cd74*^+^, and *Ryr1*^+^ cells were identified, and the distance to the nearest *Lepr*^+^ cell was manually quantified using NDP.view2 (Hamamatsu).

### Supplementary information

Figure S1

Figure S2

Figure S3

Table S1

Table S2

Table S3

Table S4

Table S5

Table S6

Table S7

Supplementary figure legends

## Data Availability

Spatial transcriptomic data generated for this paper have been deposited in the Gene Expression Omnibus (GEO) database under the accession code GSE228534.
