# Supplementary figures and images for: Spatial transcriptomic interrogation of the murine bone marrow signaling landscape
Source: Bone Res. 2023 Nov 6;11:59. doi: 10.1038/s41413-023-00298-1 (PMC10625929; doi:10.1038/s41413-023-00298-1)

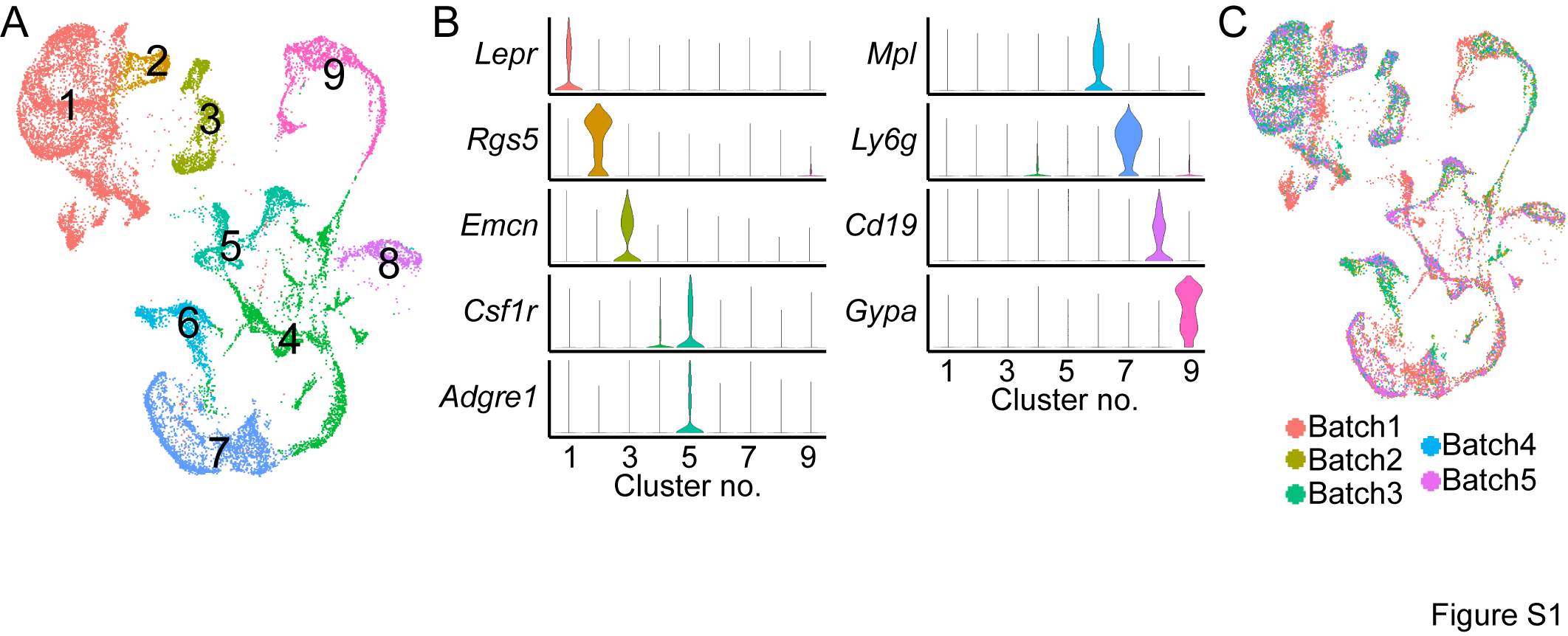

Supplement: Supplementary file 1 — Figure S1 [file 41413_2023_298_MOESM1_ESM.tif]

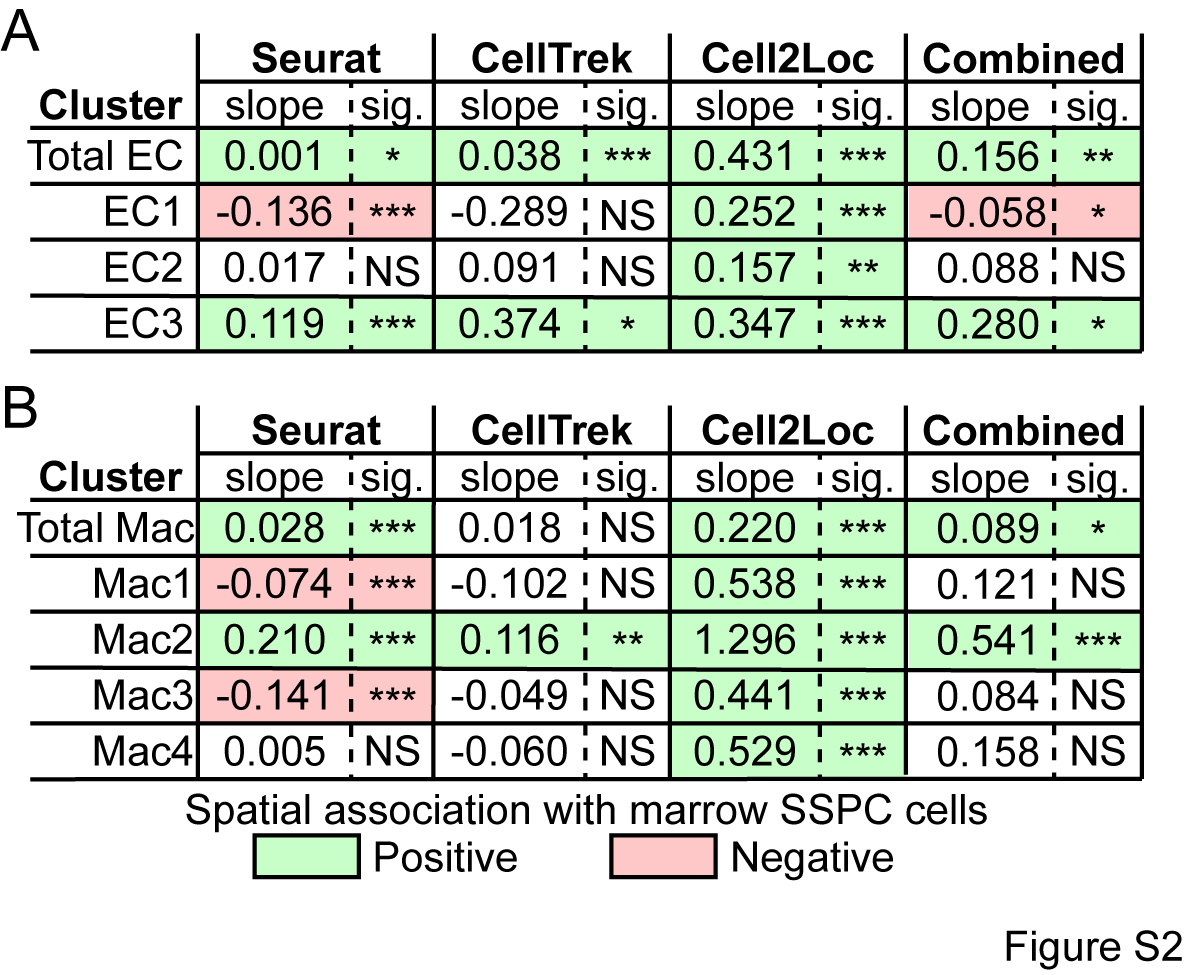

Supplement: Supplementary file 2 — Figure S2 [file 41413_2023_298_MOESM2_ESM.tif]

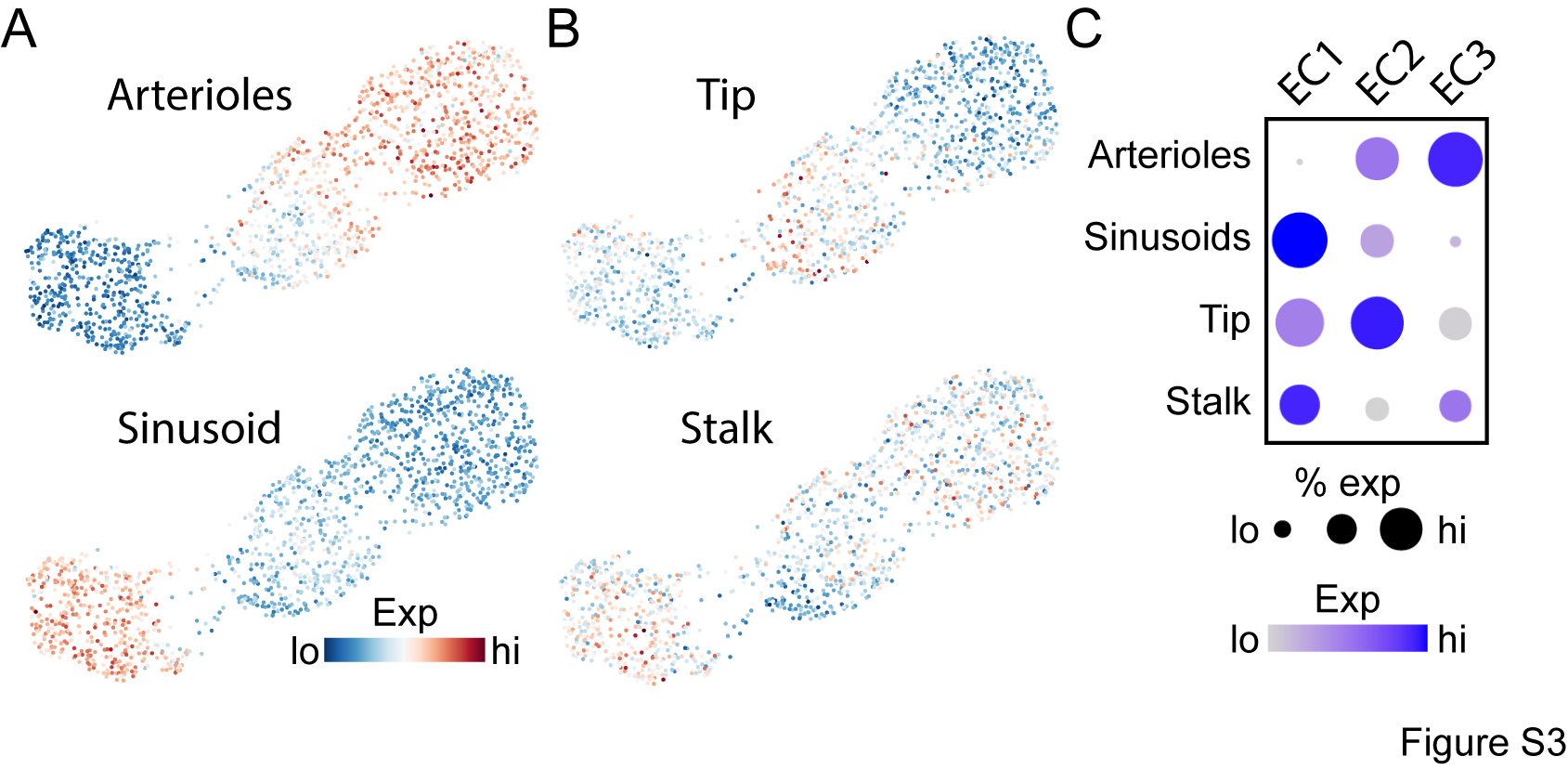

Supplement: Supplementary file 3 — Figure S3 [file 41413_2023_298_MOESM3_ESM.tif]
