# Supplementary material for: Spatial transcriptomic interrogation of the murine bone marrow signaling landscape
Source: Bone Res. 2023 Nov 6;11:59. doi: 10.1038/s41413-023-00298-1 (PMC10625929; doi:10.1038/s41413-023-00298-1)
Supplement: Supplementary file 11 — Supplementary figure legends [file 41413_2023_298_MOESM11_ESM.docx]

Figure S1 **Whole bone marrow single-cell dataset characterization**. (**A**) UMAP colored by cell cluster of the whole bone marrow merged dataset. (**B**) Representative marker genes for each cluster. (**C**) UMAP colored by batch of the whole bone marrow merged dataset. Batch 1 is derived from Zhong et al.^7^, and Batches 2-5 are derived from Tikhonova et al.^12^.

Figure S2 **SSPC niche composition of cellular subtypes**. (**A**) Correlation analyses of endothelial cell (EC) subtypes within the SSPC niche. (**B**) Correlation analyses of macrophage (Mac) subtypes within the SSPC niche. NS=not significant, *p<0.05, **p<0.01, ***p<0.001.

Figure S3 **Characterization of endothelial subtypes**. (**A**) Feature plot showing the module score for genes previously associated with either arterioles or sinusoids^26^. (**B**) Feature plot showing the module score for genes previously associated with either tip or stalk endothelial cells^27^. **(C)** Dot plot showing module scores across all EC subpopulations.
